# Multidisciplinary management of cardiovascular disease in women: Delphi consensus

**DOI:** 10.3389/fcvm.2024.1315503

**Published:** 2024-02-21

**Authors:** José M. Gámez, Milagros Pedreira Pérez, María Rosa Fernández Olmo, María Fasero Laiz, Verónica Inaraja, Vicente Pallarés Carratalá

**Affiliations:** ^1^ Servicio de Cardiología, Hospital Universitario Son Llatzer, Departamento de Medicina, Universidad de las Islas Baleares, Palma de Mallorca, Spain; ^2^ CIBER de Fisiopatología de la Obesidad y la Nutrición (CIBEROBN CB12/03/30038), Instituto de Salud Carlos III, Madrid, Spain; ^3^ Coordinadora de la Unidad de Cardio-Oncología y de la Unidad de Enfermedad Cardiovascular en la Mujer, Hospital Clínico Universitario Santiago de Compostela, A Coruña, Spain; ^4^ Especialista en Cardiología en la Unidad de Rehabilitación Cardiaca, Hospital Universitario de Jaén, Jaén, Spain; ^5^ Coordinadora de la Unidad de Menopausia Saludable, Hospital Universitario La Zarzuela, Madrid, Spain; ^6^ Unidad de Menopausia Saludable, Clínica Corofas, Madrid, Spain; ^7^ Facultad de Medicina, Universidad Francisco de Vitoria, Madrid, Spain; ^8^ Departamento Médico, Organon Salud España, Madrid, Spain; ^9^ Unidad de Vigilancia de la Salud, Unión de Mutuas de Castellón, Castellón, Spain; ^10^ Departamento de Medicina, Universitat Jaume I, Castellón, Spain

**Keywords:** cardiovascular disease, Delphi, diagnosis, women, multidisciplinary, statins

## Abstract

**Background:**

Current clinical guidelines on cardiovascular disease (CVD) do not specifically address the female population. The aim of this consensus is to know the opinion of a group of experts on the management of CVD in women.

**Methods:**

Through a Delphi consensus, 31 experts in cardiology, 9 in gynecology and obstetrics, and 14 primary care physicians, showed their degree of agreement on 44 items on CVD in women divided into the following groups: (1) risk factors and prevention strategies; (2) diagnosis and clinical manifestations; and (3) treatment and follow-up.

**Results:**

After two rounds, consensus in agreement was reached on 27 items (61.4%). Most of the non-consensus items (31.8%) belonged to group 3. The lack of consensus in this group was mainly among gynecologists and primary care physicians. The panelists agreed on periodic blood pressure control during pregnancy and delivery to detect hypertensive disorders, especially in women with a history of preeclampsia and/or gestational hypertension, and diabetes mellitus control in those with gestational diabetes. Also, the panelists agreed that women receive statins at a lower intensity than men, although there was no consensus as to whether the efficacy of drug treatments differs between women and men.

**Conclusions:**

The high degree of consensus shows that the panelists are aware of the differences that exist between men and women in the management of CVD and the need to propose interventions to reduce this inequality. The low level of consensus reveals the lack of knowledge, and the need for information and training on this topic.

## 1 Introduction

Cardiovascular disease (CVD) is the leading cause of death for both men and women in Spain (1) and worldwide (2). The prevalence for certain cardiovascular conditions, such as myocardial infarction or stroke, is higher in women (2, 3). In countries with higher socioeconomic status, the last three decades have witnessed a reduction in mortality rates related to CVD in women; however, there is still a worrying trend towards an increase in the number of myocardial infarctions, especially in young women (4). Prevalence of CVDs is lower in premenopausal women than in men of the same age, with differences reversing in the postmenopausal period (5). This ongoing change in the knowledge of CVD results in new challenges for healthcare services to provide the best care to women at all stages of their lives.

There are several reasons to explain these differences. On the one hand, women are under-represented in clinical studies, accounting for only 26%–37% of the study population (6); and, on the other hand, the parameters developed for CVD management, such as risk score scales, come from studies conducted mainly in a mostly male population (2). Also, there are pathophysiological differences in the presentation of CVD such as lower atherosclerotic and of obstructive disease burden (7, 8). In addition, in women, several sign of myocardial infarction are different from men, for example, discomfort in jaw, dyspnea or diaphoresis are more frequent in women and could lead to a delayed diagnosis (7). Finally, several studies have shown a lower frequency of treatments and procedures (such as aspirin, β-blockers, reperfusion therapy, cardiac catheterization and revascularization procedures) according to clinical guidelines for women and higher mortality rates after a coronary event (9, 10).

In recent years, healthcare trends are changing and there are several initiatives to explore possible differences in presentation, risk factors and treatment differences in women with regarding CVD (4). Women-specific cardiovascular risk factors such as premature birth, gestational hypertension or gestational diabetes, early menopause, and polycystic ovary syndrome have been identified (4). In addition to these specific sex factors, other socio-economic factors related to gender roles affect women's health outcomes more than men, such as lower educational level, lower wages, family responsibilities, and higher poverty rates (11).

Improving cardiovascular health in women requires a multidisciplinary approach that involves all healthcare professionals committed to their care throughout their lives, both in terms of their physical health and those factors that may influence their utilization of health resources. Using a Delphi methodology, the overall purpose of this work is to know the opinion of a panel of experts on the management of women with CVD and to raise awareness about the impact of sex and gender on the diagnosis and management of CVD. Unlike other consensus documents (12), this one includes primary care physicians, who are often first professional that women turn to.

## 2 Methods

### 2.1 Study design

The study used a modified Delphi method, a structured communication technique that allows a group of experts to gather opinions on a given complex or controversial topic for which there is insufficient evidence or their knowledge is incomplete or uncertain (13, 14). In addition, it allows the opinions of a group of experts to be explored and unified without the difficulties and inconveniences inherent to consensus methods based on face-to-face discussions, such as displacements or the biases of influence or non-confidential interaction.

The study was carried out between September 2022 and December 2022. It was performed in three phases: (a) Preparation of an evidence dossier to identify knowledge gaps about the management of CVD in women; (b) meeting of the scientific expert committee to review the evidence dossier and agree on the Delphi survey items; (c) two successive rounds of online surveys to gather the opinion of a panel of experts about these items; and (d) analysis and discussion of the results to draw conclusions.

### 2.2 Participants

Three types of professionals participated in the study: a scientific committee, a technical team, and a panel of experts. The scientific committee consisted of three cardiologists, one gynecologist and one primary care physician, whose role was to review the literature and draft a questionnaire with items regarding the management of CVD in women. The technical team, which directed and supervised the entire process, was responsible for the instrumental implementation of the method (search of the literature, distribution of the questionnaire to the panelists, analysis of the responses, and statistical interpretation of the consensus). Finally, the scientific committee chose a panel of experts in cardiology, gynecology and primary care according to the role and experience of these specialties in the prevention, diagnosis and treatment of women with CVD at different stages and with different ages, and according to the geographic region to which they belong, trying to achieve the maximum representation of all of Spain. The panel of experts consisted of 31 experts in cardiology, 9 in gynecology and obstetrics, and 14 in family and community medicine.

### 2.3 Delphi questionnaire

Based on the discussions of the scientific committee, and according to the evidence found on the topic, the scientific committee developed a Delphi questionnaire consisting of 44 items divided in three groups that included the most relevant controversies on the management of CVD in women: (1) risk factors and prevention strategies (16 items); (2) diagnosis and clinical manifestations of the disease (7 items); and (3) treatment and follow-up of CVD in women (21 items).

For the evaluation of the questionnaire, a single 9-point Likert-type ordinal scale was proposed, according to the model developed by the UCLA-RAND Corporation for the comparative evaluation and prioritization between different health care options (minimum 1, complete disagreement; and maximum 9, complete agreement) (14). This scale was structured in three groups according to the level of agreement-disagreement of the statement: from 1 to 3, interpreted as rejection or disagreement; from 4 to 6, interpreted as no agreement or disagreement; and from 7 to 9, interpreted as expression of agreement or support.

### 2.4 Phases of Delphi consensus

Following the Delphi methodology procedure (15), the questionnaire was sent to the panel of experts to respond by showing their degree of agreement with the items. In the 1st round, the panelists responded to the questionnaire *online* and were offered the possibility of adding their opinion as an open text. The technical team evaluated and presented the results of the 1st round using bar graphs to facilitate comments and clarifications from each participant. In the 2nd round, the panelists contrasted their personal opinion with that of the other participants and, if necessary, reconsidered their initial opinion on those items where consensus was not reached. The results of this 2nd round were tabulated and presented descriptively. In a final meeting, the scientific committee discussed and interpreted the results.

### 2.5 Analysis and interpretation of results

The median and interquartile range of the scores obtained for each item were used to analyze the data for both rounds. There was consensus when two-thirds or more of the respondents (≥66.7%) scored within the 3-point range (1–3 or 7–9) that contained the median. The type of consensus reached on each item was determined by the median score. There was agreement if the median was ≥7 and there was disagreement if the median was ≤3. No consensus was considered when one-third or more of the panelists (≥33.3%) scored in the range of 1–3 and another third or more in the range of 7–9. When the median score fell between the range of 4–6, the items were considered uncertain to a representative majority of the group.

## 3 Results

**Figure 1:**
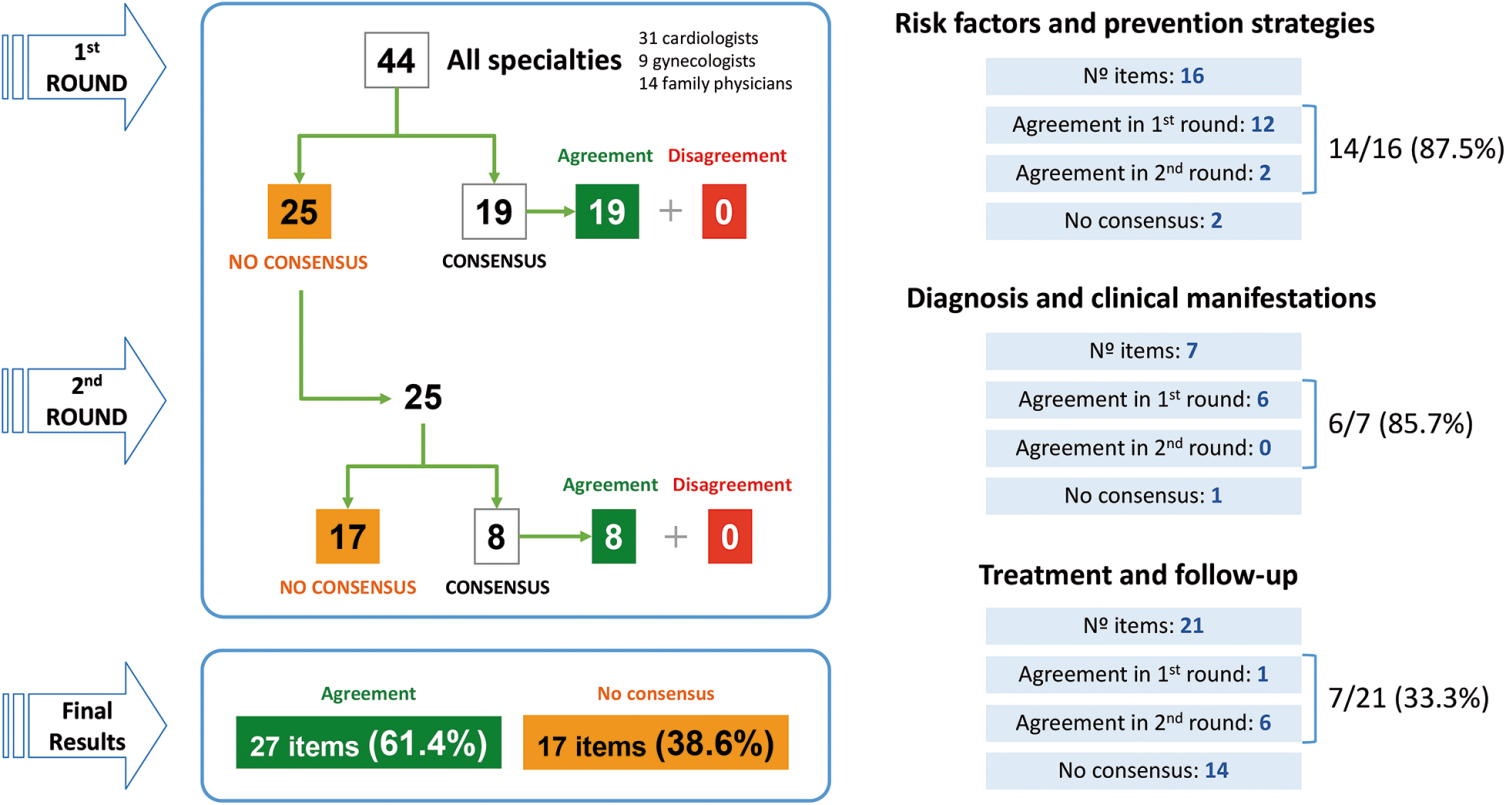
Main results of the Delphi consensus.

The 54 experts invited to participate completed the two rounds of the Delphi consensus. Of the 44 items proposed in the 1st round, consensus was reached on 19 (43.2%), all of them in agreement. The 25 non-consensus items were sent to the panelists to be assessed in a 2nd round. Of these, consensus was reached in 8 items (in agreement) and 17 did not reach consensus. After the two rounds, and a total of 44 items, 27 reached consensus in agreement (61.4%) and 17 did not reach consensus (38.6%) (Figure 1). These results differ if only expert cardiology panelists are taken into account, with 34 items in agreement (77.3%) and 10 non-consensus items (22.7%). Although results in groups 1 and 2 of the Delphi questionnaire are very similar among the different specialties of panelists, groups 3 shows a noticeable difference between cardiologists on one side and gynecologists and primary care physicians on the other side. Tables 1–3 show in detail the degree of agreement reached with each item after the two rounds. Supplementary material includes results by each specialty.

**Table 1:** Risk factors and prevention strategies.

Items	Median (IQR)	% Agreement	Round with agreement
1. Preterm delivery is an independent cardiovascular risk factor in women and, therefore, it is necessary to carry out a specific follow-up in them	8 (6–9)	74%	1st round
2. In case of preterm delivery, periodic evaluation for hypertension and diabetes mellitus, as well as other cardiovascular risk factors, should be considered	8 (7–9)	85%	1st round
3. Regular blood pressure monitoring during pregnancy and after delivery is necessary to detect hypertensive disorders of pregnancy that may lead to increased cardiovascular risk	9 (9–9)	100%	1st round
4. In women with a history of preeclampsia and/or gestational hypertension, periodic evaluation for arterial hypertension during pregnancy and after delivery should be considered	9 (9–9)	100%	1st round
5. In women with a history of gestational diabetes, periodic evaluation for diabetes mellitus during pregnancy and after delivery should be considered	9 (9–9)	100%	1st round
6. In women diagnosed with polycystic ovaries, it is recommended to monitor global cardiovascular risk	8.5 (7–9)	91%	1st round
7. Smoking is a risk factor for cardiovascular disease that affects women more than men	8 (7–9)	78%	1st round
8. Blood pressure should be controlled in the same way in men and women to reduce the impact of high blood pressure on cardiovascular events	9 (8–9)	89%	1st round
9. Although the frequency of diabetes is similar in woman and men, as a risk factor for cardiovascular disease it is more serious in women than in men	8 (7–9)	83%	**2nd round**
10. Depression is a risk factor for cardiovascular disease in women that negatively affects their prognosis	8.5 (7–9)	87%	1st round
11. Psychosocial risk factors for cardiovascular disease affect women more than men	8 (7–9)	80%	1st round
12. Women with less social support, less economic stability and less access to education and the health system than men have a higher risk of cardiovascular disease	9 (8–9)	83%	1st round
13. According to clinical guidelines, cardiovascular risk is assessed differently in men than in women	8 (6–9)	74%	**2nd round**
14. Cardiovascular disease prevention strategies currently employed in women are equal to those in men	7 (3–8)	57%	No consensus
15. Clinical guidelines for cardiovascular disease prevention do not adequately address risk assessment in women	8 (7–9)	81%	1st round
16. Mammography findings, such as microcalcifications and breast density, help assess cardiovascular disease risk and mortality	5.5 (3–8)	33%	No consensus

IQR, interquartile range; Green, consensus in agreement; Orange, no consensus.

**Table 2:** Diagnosis and clinical manifestations of the disease.

Items	Median (IQR)	% Agreement	Round with agreement
17. The clinical manifestations of cardiovascular disease are the same in men and women, but women perceive them differently	7 (2–8)	54%	No consensus
18. Signs and symptoms of infarction in women may be different than in men	9 (7–9)	83%	1st round
19. Chest tightness during a heart attack is less common in women than in men	8 (6–8)	72%	1st round
20. Although women and men have the same atherosclerotic burden, obstructive disease is usually lower in women	7 (6–8)	70%	1st round
21. The atherosclerotic burden in women is more diffuse, whereas in men it is more localized	7.5 (6–8)	72%	1st round
22. INOCA (ischemia without obstructive coronary artery disease) and MINOCA (myocardial infarction in the absence of obstructive coronary artery disease) are more frequent in women than in men	8 (7–9)	85%	1st round
23. Diagnosis based on the detection of epicardial coronary stenosis is less efficient in women than in men, which leads to a worse diagnostic and therapeutic evaluation	8 (6–9)	74%	1st round

IQR, interquartile range; Green, consensus in agreement; Orange, no consensus.

**Table 3:** Treatment and follow-up of cardiovascular disease in women.

Items	All specialties			Cardiology		
	Median (IQR)	% Agreement	Round with agreement	Median (IQR)	% Agreement	Round with agreement
24. Women are underrepresented in clinical studies, clinical guidelines on treatments, and prevention and rehabilitation programs for acute coronary syndrome	9 (8–9)	91%	1st round	9 (9–9)	100%	1st round
25. In the presence of an acute coronary syndrome, fewer tests are usually performed in women than in men	8 (3–8)	63%	No consensus	8 (5–9)	71%	1st round
26. In the presence of an acute coronary syndrome, the pharmacological treatments used in women are different from those used in men	4.5 (2–8)	11%	No consensus	6 (2–8)	10%	No consensus
27. In the presence of an acute coronary syndrome, fewer coronary interventions are performed in women than in men	8 (5–9)	67%	No consensus	8 (7–9)	77%	1st round
28. In the presence of an acute coronary syndrome, reperfusion is performed later in women than in men	8 (7–9)	80%	**2nd round**	8 (8–9)	87%	1st round
29. Morbidity-mortality outcomes of coronary surgery are worse in women than in men	7 (5–9)	65%	No consensus	7 (5–9)	68%	**2nd round**
30. The therapeutic strategies used in women for acute coronary syndrome are less aggressive than in men	7.5 (5–9)	65%	No consensus	8 (7–9)	81%	**2nd round**
31. Women are prescribed with statins less frequently than men	7.5 (4–8)	63%	No consensus	8 (4–9)	61%	No consensus
32. Women receiving statins are older than men	8 (7–9)	78%	**2nd round**	8 (5–9)	71%	**2nd round**
33. Women receive statins at a lower intensity than men	8 (7–9)	81%	**2nd round**	8 (7–9)	77%	1st round
34. Women receive less combined lipid-lowering treatments (statins + ezetimibe) than men	8 (5–8)	67%	No consensus	7 (5–9)	65%	No consensus
35. The efficacy of pharmacological treatments is the same in men as in women	7.5 (5–9)	63%	No consensus	7 (5–9)	58%	No consensus
36. The efficacy of coronary interventions is the same in men as in women	8 (5–9)	69%	**2nd round**	8 (4–9)	68%	**2nd round**
37. The efficacy of statins in primary prevention is greater in women than in men	5 (2–7)	31%	No consensus	5 (3–8)	32%	No consensus
38. Cardiac rehabilitation is prescribed less in women than in men	8 (7–9)	81%	**2nd round**	9 (8–9)	90%	**2nd round**
39. Compliance with cardiac rehabilitation appointments is lower in women than in men	8 (4–9)	57%	No consensus	8 (5–9)	74%	1st round
40. Compliance with physical exercise in women after a cardiac rehabilitation program is higher than in men	7 (5–8)	59%	No consensus	7 (3–8)	58%	No consensus
41. After an acute myocardial infarction, women receive less pharmacological treatment than men	7 (2–8)	54%	No consensus	7 (5–9)	68%	1st round
42. After an acute myocardial infarction, fewer coronary interventions are performed in women than in men	7 (5–8)	63%	No consensus	8 (5–9)	74%	**2nd round**
43. After an acute myocardial infarction, the results obtained with coronary intervention are worse in women than in men	6 (2–8)	19%	No consensus	6 (2–8)	16%	No consensus
44. Mortality after a coronary event is higher in women than in men	8 (7–9)	80%	**2nd round**	8 (7–9)	90%	**2nd round**

IQR, interquartile range; Green, consensus in agreement; Orange, no consensus.

### 3.1 Risk factors and disease prevention strategies

This group of items shows a high level of agreement among panelists (Table 1). Of the 16 items proposed, 14 reached consensus in agreement, most of them in the first round. Only two items were agreed in the second round. Three of the items achieved a full consensus among all panelists (items 3, 4 and 5). The panelists agreed that regular blood pressure monitoring during pregnancy and after delivery is necessary to detect hypertensive disorders, especially in women with a history of preeclampsia and/or gestational hypertension. In addition, they agreed that periodic evaluation for diabetes mellitus during pregnancy and after delivery should be considered in women with history of gestational diabetes.

The two items that remained without consensus stated that CVD prevention strategies currently employed in women are equal to those in men, and that mammography findings, such as microcalcifications and breast density, help to assess CVD risk and mortality.

### 3.2 Diagnosis and clinical manifestations of the disease

Of the 7 items proposed, only one remained without consensus (Table 2). This item said that the clinical manifestations of CVD are the same in men and women, but women perceive them differently.

Three of the items with the higher degree of consensus stated that: signs and symptoms of infarction in women may be different to that in men (item 18, 83% of agreement); ischemia without obstructive coronary artery disease (INOCA) and myocardial infarction in the absence of obstructive coronary artery disease (MINOCA) are more frequent in women than in men (item 22, 85% of agreement); and diagnosis based on the detection of epicardial coronary stenosis is less efficient in women than in men, which leads to a worse diagnostic and therapeutic evaluation (item 23, 74% of agreement).

### 3.3 Treatment and follow-up of cardiovascular disease in women

This group of items showed the lowest level of agreement (Table 3). Even more, results differed significantly between cardiologists and the other two specialties (gynecologists and primary care physicians). Considering all specialties together, of the 21 items proposed only 7 were agreed (most of them in the second round) and 14 did not achieved consensus. On the contrary, considering only the responses of cardiologists, 14 items were agreed and 7 remained without consensus.

Of the 21 items in this group, 7 were agreed by all specialties. Some of them stated that women are underrepresented in clinical studies, clinical guidelines on treatments, and prevention and rehabilitation programs for acute coronary syndrome (item 24, 91% agreement all, 100% agreement cardiologists); women receive statins at a lower intensity than men (item 33, 81% agreement all, 77% agreement cardiologists); and mortality after a coronary event is higher in women than in men (item 44, 80% agreement all, 90% agreement cardiologists).

On the other hand, 7 items did not achieved consensus considering all panelists. Some of these non-consensus items said that, in comparison with men, women receive different pharmacological treatments in the presence of an acute coronary syndrome (item 26), are prescribed with statins less frequently (item 31), received less combined lipid-lowering treatments (item 34), and comply better with physical exercise after a cardiac rehabilitation program (item 40). In addition, panelists also did not reach an agreement about that the efficacy of pharmacological treatments is the same in women and men (item 35), that the efficacy of statins in primary prevention is greater in women than in men (item 37), and that the results with a coronary intervention after an acute myocardial infarction are worse in women than in men (item 43).

Of note are the remaining 7 items that were only agreed among cardiologists. These specialists agreed that in the presence of an acute coronary syndrome, fewer tests (item 25, 71% agreement) and fewer coronary interventions (item 27, 77% agreement) are usually performed in women than in men. They also agreed that after an acute myocardial infarction, women receive less pharmacological treatments than men (item 41, 68%) and fewer coronary interventions are performed in women (item 42, 74%). Other items agreed by cardiologists stated that morbidity-mortality outcomes of coronary surgery are worse in women (item 29, 68%), and that the therapeutic strategies used in women for acute coronary syndrome are less aggressive than in men (item 30, 81%).

## 4 Discussion

The results of this Delphi consensus show the differences in the management of CVD in women by the three participating specialties. However, the level of agreement reached on risk factors, prevention strategies, diagnosis, treatment, and follow-up (61.4%) reveals that the panelists are aware of the differences among women and men. On the other hand, the low level of consensus on treatment and follow-up evidences the lack of knowledge on these topics, as they did not reach consensus in most of the items proposed (38.6%).

### 4.1 Risk factors and disease prevention strategies

Although all the panelists agreed with most of the items related to risk factors and prevention strategies, there were two items on which consensus was not reached.

One of the non-consensus items stated that prevention strategies are the same in men and women. However, analyzing by specialties, only primary care physicians agreed with the equity of these strategies. The other specialties showed a great diversity of opinions between those who considered that these strategies should be the same and those who considered that they should be adapted to the circumstances of each sex. Regarding the latter, some panelists explained that many of the prevention strategies used in women do not take into account sex-specific risk factors. As described in some publications, the use of sex- and age-specific CVD risk thresholds and the incorporation of new measures of subclinical disease (e.g., coronary calcium score) in risk assessment could improve the targeting of preventive measures (11, 16). Indeed, a study that evaluated physician adherence to CVD prevention guidelines showed that primary care physicians, cardiologists and gynecologists were more likely to assign a lower category risk from the Framingham risk score to women at intermediate CVD risk (17).

However, how to include and account for sex-specific and underrecognized factors in risk calculators remains uncertain, and women may be particularly affected by these limitations of current risk prediction tools. Although some guidelines point to the limited evidence on the role of sex-specific factors in predicting cardiovascular risk and disease (18), others mention premature menopause and pregnancy-related disorders as factors that increase risk (19, 20). A consensus document from cardiologists, gynecologists, and endocrinologists from the European Society of Cardiology shows gynecological and obstetric conditions that interact with cardiovascular risk in women, including pregnancy disorders (recurrent pregnancy loss, preterm delivery, small-for-gestational-age, hypertensive pregnancy disorders, gestational diabetes), menopause (high adiposity, high insulin resistance, pro-atherogenic lipid profile, high heart rate variability), and other endocrine and gynecological conditions (polycystic ovarian syndrome, hypogonadotrophic hypogonadism, premature ovarian insufficiency, endometriosis) (12). Another consensus document from the Cardiology, Gynecology and two Primary Care Spanish Societies includes recommendations to improve women's follow up after having cardiovascular and metabolic complications during pregnancy (21). This document highlights the importance of primary care physicians in implementing and sustaining prevention strategies to reduce the cardiovascular risk of these women.

The second non-consensus item (by none of the participating specialties), stated that mammography findings, such as microcalcifications or breast density, can help assess CVD risk and mortality. The panelists commented that they were unaware of it. In fact, this is a finding in a recent study involving 57,867 women who prospectively underwent mammography. The authors found that mammographic features are associated with cardiometabolic risk and mortality (22). On the one hand, they found that a higher number of microcalcifications were associated with increased risk for multiple cardiometabolic diseases and an increased cardiometabolic mortality, mainly in women with pre-existing cardiometabolic diseases. On the other hand, dense breasts were associated with a lower incidence of cardiometabolic diseases (22). These findings could help assess the cardiovascular risk of women during routine mammography.

### 4.2 Diagnosis and clinical manifestations of the disease

The majority of the proposed items on the diagnosis and clinical manifestations of CVD were agreed by all the participating specialties. They considered that signs and symptoms of CVD are different in women and men. The only item that was not agreed by all the specialties stated that the clinical manifestations of CVD are the same in men and women, but women perceive these manifestations differently as previously described (23). Although most of the panelists agreed that women tend to perceive these manifestations differently from men, there was no clear agreement on whether or not the manifestations are really the same between men and women. Considering that a clinical manifestation is the relationship between the signs and symptoms that occur in a patient, although the signs and symptoms between men and women are different, the same manifestations could occur (7, 8, 24). Hence the great diversity of opinions among the panelists. In fact, when asked directly about signs and symptoms, all the panelists agreed that there are differences between men and women. This diversity of opinions, together with different perception of clinical manifestations by women, can delay the diagnosis and even the appropriate treatment of the disease (23).

### 4.3 Treatment and follow-up of cardiovascular disease in women

The items on the treatment and follow-up of CVD in women showed the great difference between cardiologists and the other specialties. While cardiologists agreed most of the items, either in the first or second round, gynecologists and primary care physicians did not reach consensus on most of them, which shows a significant lack of knowledge on the topic. These two specialties commented that they were really unfamiliar with the treatments and tests used for CVD, since this was a field outside their specialty. Hence the importance of multidisciplinary teams to ensure continuity of care from diagnosis to treatment and subsequent follow-up. Taking into account that the treatment of these diseases is more specific to cardiologists, we performed a specific analysis of the results in this specialty.

In contrast to gynecologists and primary care physicians, cardiologists agreed that women undergo less tests or coronary interventions, and receive less pharmacological treatments than men for an acute coronary syndrome. However, cardiologists, gynecologists, and primary care physicians did not have a consensus opinion about the prescription and efficacy of statins or combined lipid-lowering treatments (statins + ezetimibe) in women. Some of the comments offered by the panelists on the use of statins said that the lower prescription of these drugs in women could be because women are better compliant with lifestyle changes than men, or because women receive statins of lower intensity or at a lower dose than men. In fact, these comments are in accordance with the agreement shown in other items, in which it was stated that women receive statins at a lower intensity than men.

Despite physiological differences in lipid metabolism in women and men, existing data suggest that statins and other lipid-lowering drugs are equally effective in women and men for both primary and secondary prevention. Of note, these studies included more men than women (25). The proportional reductions per mmol/L of LDL-cholesterol reduction in major vascular events, major coronary events, coronary revascularization, and stroke are similar in women and men (26, 27). In addition, the relative effects of drugs other than statins that lower LDL-cholesterol (ezetimibe and PCSK9 inhibitors, in addition to high-intensity statin therapy) are also similar in both women and men (26, 27). However, a recent study that evaluated the sex-specific relation between statin treatment and survival and the additional benefit of high-intensity statins showed that statins seem to be effective regardless of treatment intensity, especially in women (28). The protective effect of primary prevention statins was stronger in women than men for both all-cause and cardiovascular mortality, and high-intensity statins conferred a modest additional benefit in both sexes (28). Thus, further research and analysis of efficacy data by sex is needed, including more women in clinical trials.

The underuse of statins for both primary and secondary prevention of CVD in women compared with men is due to several factors. On the one hand, the literature shows that healthcare professionals are less likely to prescribe statins for women (mainly of high-intensity) or to follow guideline-recommended statin intensity. On the other hand, women are more likely to refuse or stop statin therapy, that is, they have a low rate of non-adherence. In addition, other factors include underestimation of cardiovascular risk, limited treatment options in pregnancy, and worse side effect profile (25, 29–32). International guidelines on the treatment of dyslipidemia largely lack specific recommendations for women (25).

In any case, and despite the differences and lack of consensus shown among the panelists, they all agreed that mortality after a coronary event is higher in women than in men. However, according to the literature, women with acute coronary syndrome living in countries of high socioeconomic status appear to have a lower risk of mortality after discharge than men. This risk is attenuated in countries with lower socioeconomic status, where adjusted mortality rates are similar between women and men (33).

### 4.4 Strengths and limitations

Despite the benefits of this methodology, this work has some limitations. The selection of panelists was neither systematic nor randomized and the ratio of specialties was not balanced; recruitment was based on their clinical expertise in the management of CVD and the role of the different specialties in the prevention, diagnoses and treatment of CVD in women. The panelists were recruited from different Spanish regions, although not all regions were represented. Finally, the results may have been influenced by ambiguity in the phrasing of some of the items. The fact that the survey was online had the advantage of anonymity, but it could lead to erroneous interpretations of the items by the panelists, which could influence the result.

## 5 Conclusions

The high degree of consensus shows that the panelists are aware of the differences that exist between men and women in the management of CVD and the need to propose interventions to reduce this inequality. However, the low level of consensus, mainly in the treatment and follow-up, reveals the lack of knowledge on this topic. For this reason, it is necessary to reinforce awareness of this issue, especially among gynecologists and primary care physicians, but also among cardiologists, highlighting the role all specialties can play in helping to promote prevention strategies, especially primary prevention. While gynecologists need to take into account situations such as pregnancy, delivery or menopause, primary care physicians, who are often the first point of contact, should be especially aware of CVD prevention strategies after delivery and during menopause. An adequate transition from gynecology to primary care after pregnancy is necessary, as well as collaboration between nursing from both specialties, accompanied by a greater awareness of professionals. Among cardiologists it is important to ensure that every women with signs and symptoms of an acute coronary syndrome is correctly diagnosed and treated. This will avoid delays in both the diagnosis and treatment of these diseases in women.

Based on the discussions that took place and the results of this Delphi consensus, the scientific committee summarizes the following agreements to improve CVD management on women:

• It is necessary to carry out a specific cardiovascular risk follow-up in women with preterm labor, preeclampsia and/or gestational hypertension, and gestational diabetes.
• A global cardiovascular risk monitoring is recommended in women diagnosed with polycystic ovaries.
• Tobacco, diabetes, depression, and psychosocial condition are risk factors for cardiovascular disease that should be specially considered in the cardiovascular evaluation in women (as they affect more them).
• Clinical guidelines for the prevention of CVD should address risk assessment in women.
• There is a need on educational awareness to health care providers and general population about the differences in signs and symptoms of a heart attack in women.
• As the atherosclerotic burden in women is more diffuse, the detection should not be restricted to epicardial coronary.
• Women should be included in clinical studies, clinical guidelines on treatment, prevention, and rehabilitation programs for acute coronary syndrome.
• When women have signs or symptoms of a potential acute coronary syndrome or myocardial infarction, there should be assessed the same tests as if it was a men.
• The therapeutic strategies used for acute coronary syndrome or myocardial infarction in women should be the same as in men.
• Cardiac rehabilitation should be prescribed in women as in men.

## Data Availability

The original contributions presented in the study are included in the article/**Supplementary Material**, further inquiries can be directed to the corresponding author.

## Appendix

Panel of experts participating in the Delphi consensus.

**Table:**

Specialty	Expert	Hospital
Cardiology	Adriana Saltijeral Cerezo	Hospital Vithas Aravaca.Madrid
	Alberto Cordero Fort	Hospital Universitario de San Juan. Alicante
	Almudena Aguilera Saborido	Hospital Universitario Virgen del Rocío. Sevilla
	Almudena Castro Conde	Hospital Universitario La Paz. Madrid
	Ana Belén Cid Álvarez	Hospital Clínico Universitario de Santiago de Compostela
	Carlos Escobar Cervantes	Hospital Universitario La Paz. Madrid
	Carmen Rus Mansilla	Hospital Alto Guadalquivir. Jaén
	Clara Bonanad	Hospital Clínico Universitario de Valencia
	Concepción Alonso Martín	Hospital de La Santa Creu i Sant Pau. Barcelona
	David González Calle	Hospital Universitario de Salamanca
	Dolores Mesa Rubio	Hospital Universitario Reina Sofía. Córdoba
	Eva María Pereira López	Hospital Universitario Lucus Augusti. Lugo
	Gabriela Guzmán Martínez	Hospital Universitario La Paz/Universidad Europea de Madrid/ATRYS
	Irene Rilo Miranda	Hospital Universitario Donostia
	Javier Mora Robles	Hospital Regional Universitario de Málaga
	Juan Miguel Ruiz Nodar	Hospital General Universitario de Alicante
	Laura Quintas Ovejero	OSI Bajo Deba. Guipúzcoa
	Mª Pilar Portero Pérez	Hospital San Pedro. Logroño
	María del Mar Martínez Quesada	Hospital Virgen Macarena. Sevilla
	María del Rosario Ortas Nadal	Hospital Universitario Miguel Servet. Zaragoza
	Marta Aliacar Muñoz	Hospital Royo Villanova. Zaragoza
	Miriam Auxiliadora Martín Toro	Hospital Universitario de Puerto Real. Cádiz
	Miriam Sandín Rollán	Hospital General Universitario de Alicante
	Nieves Romero Rodríguez	Hospital Universitario Virgen del Rocío. Sevilla
	Pilar Jiménez Quevedo	Hospital Clínico San Carlos. Madrid
	Pilar Mazón Ramos	Hospital Clínico Universitario de Santiago de Compostela
	Raquel Campuzano	Hospital Universitario Fundación de Alcorcón. Madrid
	Regina Dalmau González-Gallarza	Hospital Universitario La Paz. Madrid
	Román Freixa Pamias	Complex Hospitalari Moisès Broggi. Barcelona
	Vicente Ignacio Arrarte Esteban	Hospital General Universitario Dr. Balmis. Alicante
	1 expert	Hospital Universitario de Navarra
Gynecology and Obstetrics	Alicia Hernández Gutiérrez	Hospital Universitario La Paz. Madrid
	Ana Isabel Prieto Amorín	Hospital de La Zarzuela. Madrid
	Belén Santacruz Martín	Hospital Universitario de Torrejón. Madrid
	Carmen Pingarrón Santofimia	Hospital Quironsalud San José. Madrid
	Isabel Gippini Requeijo	Hospital Campo Grande. Valladolid
	María Jesús Cancelo Hidalgo	Hospital Universitario de Guadalajara. Universidad de Alcalá
	Mercedes Andeyro García	Hospital Universitario General de Villalba. Madrid
	Mercedes Herrero Conde	Hospital HM Montepríncipe y HM Sanchinarro. Madrid
	Virginia Díaz Miguel	Hospital Universitario del Henares. Madrid
Primary care physician	Ana M Piera Carbonell	Centro de Salud La Corredoria. Oviedo. Área IV. Sespa
	Ana Moyá Amengual	Centro de Salud Sta. Catalina. Palma de Mallorca
	Ángel Vicente Molinero	Centro de Salud Utebo. Zaragoza
	Anny Romero Secin	Consultorio Colloto. Oviedo
	Antonio Ruiz García	Centro de Salud Universitario Pinto. Madrid
	Ignacio González Lillo	Centro de Salud Picarral. Zaragoza
	Laura Aparisi Esteve	Centro de Salud Carinyena, Vilarreal. Castellón
	Lisardo García Matarín	Aguadulce Sur. Almería
	María Inmaculada Cervera Pérez	Consultorio Tendetes. Valencia
	María Isabel Hervella Durántez	Centro de Salud Calle Gerona. Alicante
	María José Castillo Moraga	Centro de Salud Barrio Bajo. Sanlúcar de Barrameda. Cádiz
	Miguel Turégano Yedro	Centro de Salud Aldea Moret. Cáceres
	Rafael Crespo Sabarís	Atención Primaria La Rioja
	1 expert	Centro de Salud Muro de Alcoy. Alicante
