# Supplementary material for: Multidisciplinary management of cardiovascular disease in women: Delphi consensus
Source: Front Cardiovasc Med. 2024 Feb 21;11:1315503. doi: 10.3389/fcvm.2024.1315503 (PMC10914989; doi:10.3389/fcvm.2024.1315503)
Supplement: Supplementary file 1 [file Datasheet1.docx]

Supplementary Material

**Table S1**. Risk factors and prevention strategies

| **Items** | **All specialties** | | **Gynecology** | | **Primary Care Physicians** | | **Cardiology** | |
| --- | --- | --- | --- | --- | --- | --- | --- | --- |
|  | **Median**  **(IQR)** | **% Agreement** | **Median**  **(IQR)** | **% Agreement** | **Median**  **(IQR)** | **% Agreement** | **Median**  **(IQR)** | **% Agreement** |
| 1. Preterm delivery is an independent cardiovascular risk factor in women and, therefore, it is necessary to carry out a specific follow-up in them. | 8 (6-9) | 74% | 6 (4-8.5) | 33% | 8 (8-9) | 86% | 7 (7-9) | 77% |
| 1. In case of preterm delivery, periodic evaluation for hypertension and diabetes mellitus, as well as other cardiovascular risk factors, should be considered. | 8 (7-9) | 85% | 8 (6-9) | 78% | 8.5 (8-9) | 100% | 8 (7-9) | 81% |
| 1. Regular blood pressure monitoring during pregnancy and after delivery is necessary to detect hypertensive disorders of pregnancy that may lead to increased cardiovascular risk. | 9 (9-9) | 100% | 9 (9-9) | 100% | 9 (9-9) | 100% | 9 (9-9) | 100% |
| 1. In women with a history of preeclampsia and/or gestational hypertension, periodic evaluation for arterial hypertension during pregnancy and after delivery should be considered. | 9 (9-9) | 100% | 9 (9-9) | 100% | 9 (9-9) | 100% | 9 (9-9) | 100% |
| 1. In women with a history of gestational diabetes, periodic evaluation for diabetes mellitus during pregnancy and after delivery should be considered. | 9 (9-9) | 100% | 9 (9-9) | 100% | 9 (9-9) | 100% | 9 (9-9) | 100% |
| 1. In women diagnosed with polycystic ovaries, it is recommended to monitor global cardiovascular risk. | 8.5 (7-9) | 91% | 9 (7-9) | 78% | 8.5 (7-9) | 100% | 8 (7-9) | 90% |
| 1. Smoking is a risk factor for cardiovascular disease that affects women more than men. | 8 (7-9) | 78% | 9 (6-9) | 78% | 7.5 (6-9) | 64% | 8 (7-9) | 84% |
| 1. Blood pressure should be controlled in the same way in men and women to reduce the impact of high blood pressure on cardiovascular events. | 9 (8-9) | 89% | 9 (7-9) | 78% | 8 (8-9) | 79% | 9 (9-9) | 97% |
| 1. Although the frequency of diabetes is similar in woman and men, as a risk factor for cardiovascular disease it is more serious in women than in men. | 8 (7-9) | 83% | 8 (7-9) | 89% | 7.5 (5-8) | 64% | 8 (8-9) | 90% |
| 1. Depression is a risk factor for cardiovascular disease in women that negatively affects their prognosis. | 8.5 (7-9) | 87% | 8 (4.5-8.5) | 67% | 9 (7-9) | 93% | 9 (8-9) | 90% |
| 1. Psychosocial risk factors for cardiovascular disease affect women more than men. | 8 (7-9) | 80% | 8 (6.5-9) | 78% | 8 (7-9) | 86% | 8 (7-9) | 77% |
| 1. Women with less social support, less economic stability and less access to education and the health system than men have a higher risk of cardiovascular disease. | 9 (8-9) | 83% | 8 (6.5-8.5) | 78% | 9 (7-9) | 79% | 9 (8-9) | 87% |
| 1. According to clinical guidelines, cardiovascular risk is assessed differently in men than in women. | 8 (6-9) | 74% | 7 (5-9) | 67% | 8 (8-9) | 93% | 8 (2-8) | 68% |
| 1. Cardiovascular disease prevention strategies currently employed in women are equal to those in men. | 7 (3-8) | 57% | 7 (3-8) | 67% | 7.5 (6-9) | 71% | 6 (3-8) | 13% |
| 1. Clinical guidelines for cardiovascular disease prevention do not adequately address risk assessment in women. | 8 (7-9) | 81% | 8 (4.5-9) | 56% | 7.5 (7-9) | 79% | 8 (7-9) | 90% |
| 1. Mammography findings, such as microcalcifications and breast density, help assess cardiovascular disease risk and mortality. | 5.5 (3-8) | 33% | 5 (2-75) | 22% | 5.5 (4-7) | 50% | 6 (3-8) | 29% |

IQR: interquartile range.

Green: consensus in agreement; Orange: No consensus.

**Table S2**. Diagnosis and clinical manifestations of the disease

| **Items** | **All specialties** | | **Gynecology** | | **Primary Care Physicians** | | **Cardiology** | |
| --- | --- | --- | --- | --- | --- | --- | --- | --- |
|  | **Median**  **(IQR)** | **% Agreement** | **Median**  **(IQR)** | **% Agreement** | **Median**  **(IQR)** | **% Agreement** | **Median**  **(IQR)** | **% Agreement** |
| 1. The clinical manifestations of cardiovascular disease are the same in men and women, but women perceive them differently. | 7 (2-8) | 54% | 7 (2.5-7.5) | 67% | 7.5 (2-8) | 64% | 6 (2-8) | 16% |
| 1. Signs and symptoms of infarction in women may be different than in men. | 9 (7-9) | 83% | 8 (4.5-9) | 67% | 8 (8-9) | 86% | 9 (8-9) | 87% |
| 1. Chest tightness during a heart attack is less common in women than in men. | 8 (6-8) | 72% | 6 (4.5-8.5) | 56% | 8 (7-8) | 86% | 8 (7-9) | 77% |
| 1. Although women and men have the same atherosclerotic burden, obstructive disease is usually lower in women. | 7 (6-8) | 70% | 7 (6-8) | 56% | 6.5 (5-8) | 50% | 7 (7-8) | 84% |
| 1. The atherosclerotic burden in women is more diffuse, whereas in men it is more localized. | 7.5 (6-8) | 72% | 7 (5-7.5) | 56% | 8 (5-8) | 57% | 8 (7-9) | 84% |
| 1. INOCA (ischemia without obstructive coronary artery disease) and MINOCA (myocardial infarction in the absence of obstructive coronary artery disease) are more frequent in women than in men. | 8 (7-9) | 85% | 7 (5-8.5) | 67% | 7.5 (5-9) | 64% | 9 (8-9) | 100% |
| 1. Diagnosis based on the detection of epicardial coronary stenosis is less efficient in women than in men, which leads to a worse diagnostic and therapeutic evaluation. | 8 (6-9) | 74% | 5 (5-8) | 67% | 7 (6-8) | 64% | 9 (7-9) | 90% |

IQR: interquartile range.

Green: consensus in agreement; Orange: No consensus.

**Table S3**. Treatment and follow-up of cardiovascular disease in women

| **Items** | **All specialties** | | **Gynecology** | | **Primary Care Physicians** | | **Cardiology** | |
| --- | --- | --- | --- | --- | --- | --- | --- | --- |
|  | **Median**  **(IQR)** | **% Agreement** | **Median**  **(IQR)** | **% Agreement** | **Median**  **(IQR)** | **% Agreement** | **Median**  **(IQR)** | **% Agreement** |
| 1. Women are underrepresented in clinical studies, clinical guidelines on treatments, and prevention and rehabilitation programs for acute coronary syndrome. | 9 (8-9) | 91% | 8 (5.5-9) | 67% | 9 (7-9) | 86% | 9 (9-9) | 100% |
| 1. In the presence of an acute coronary syndrome, fewer tests are usually performed in women than in men. | 8 (3-8) | 63% | 7 (1.5-8) | 56% | 7 (5-8) | 50% | 8 (5-9) | 71% |
| 1. In the presence of an acute coronary syndrome, the pharmacological treatments used in women are different from those used in men. | 4.5 (2-8) | 11% | 5 (1.5-7.5) | 22% | 3 (2-7) | 64% | 6 (2-8) | 10% |
| 1. In the presence of an acute coronary syndrome, fewer coronary interventions are performed in women than in men. | 8 (5-9) | 67% | 8 (1.5-8) | 56% | 6 (5-9) | 36% | 8 (7-9) | 77% |
| 1. In the presence of an acute coronary syndrome, reperfusion is performed later in women than in men. | 8 (7-9) | 80% | 8 (5-8.5) | 67% | 8 (6-9) | 71% | 8 (8-9) | 87% |
| 1. Morbidity-mortality outcomes of coronary surgery are worse in women than in men. | 7 (5-9) | 65% | 7 (3.5-8.5) | 56% | 7 (5-8) | 64% | 7 (5-9) | 68% |
| 1. The therapeutic strategies used in women for acute coronary syndrome are less aggressive than in men. | 7.5 (5-9) | 65% | 5 (1.5-8) | 22% | 5.5 (5-8) | 43% | 8 (7-9) | 81% |
| 1. Women are prescribed with statins less frequently than men. | 7.5 (4-8) | 63% | 5 (1.5-7.5) | 22% | 8 (7-8) | 79% | 8 (4-9) | 61% |
| 1. Women receiving statins are older than men. | 8 (7-9) | 78% | 8 (7-8) | 89% | 8 (7-9) | 86% | 8 (5-9) | 71% |
| 1. Women receive statins at a lower intensity than men. | 8 (7-9) | 81% | 8 (6-8) | 78% | 8 (7-9) | 93% | 8 (7-9) | 77% |
| 1. Women receive less combined lipid-lowering treatments (statins + ezetimibe) than men. | 8 (5-8) | 67% | 8 (5-8) | 67% | 7.5 (6-8) | 71% | 7 (5-9) | 65% |
| 1. The efficacy of pharmacological treatments is the same in men as in women. | 7.5 (5-9) | 63% | 7 (5-9) | 67% | 8 (5-9) | 71% | 7 (5-9) | 58% |
| 1. The efficacy of coronary interventions is the same in men as in women. | 8 (5-9) | 69% | 8 (7-9) | 89% | 7.5 (5-8) | 57% | 8 (4-9) | 68% |
| 1. The efficacy of statins in primary prevention is greater in women than in men. | 5 (2-7) | 31% | 2 (2-5) | 67% | 5 (2-5) | 36% | 5 (3-8) | 32% |
| 1. Cardiac rehabilitation is prescribed less in women than in men. | 8 (7-9) | 81% | 7 (5-8.5) | 67% | 7 (5-9) | 71% | 9 (8-9) | 90% |
| 1. Compliance with cardiac rehabilitation appointments is lower in women than in men. | 8 (4-9) | 57% | 5 (2.5-8.5) | 33% | 5 (4-8) | 50% | 8 (5-9) | 74% |
| 1. Compliance with physical exercise in women after a cardiac rehabilitation program is higher than in men. | 7 (5-8) | 59% | 8 (6-8.5) | 78% | 6.5 (3-8) | 50% | 7 (3-8) | 58% |
| 1. After an acute myocardial infarction, women receive less pharmacological treatment than men. | 7 (2-8) | 54% | 7 (2-8) | 56% | 5 (2-6) | 36% | 7 (5-9) | 68% |
| 1. After an acute myocardial infarction, fewer coronary interventions are performed in women than in men. | 7 (5-8) | 63% | 8 (2-8.5) | 56% | 5.5 (3-8) | 29% | 8 (5-9) | 74% |
| 1. After an acute myocardial infarction, the results obtained with coronary intervention are worse in women than in men. | 6 (2-8) | 19% | 5 (2-7) | 11% | 6 (2-7) | 29% | 6 (2-8) | 16% |
| 1. Mortality after a coronary event is higher in women than in men. | 8 (7-9) | 80% | 8 (4.5-9) | 78% | 7 (2-8) | 57% | 8 (7-9) | 90% |

IQR: interquartile range.

Green: consensus in agreement; Orange: No consensus.
